# Obsessive Beliefs in Posttraumatic Stress Disorder

**DOI:** 10.1007/s10942-025-00597-y

**Published:** 2025-06-26

**Authors:** Robert E. Fite, Johanna Thompson-Hollands

**Affiliations:** 1 National Center for PTSD at VA Boston Healthcare System, Boston, MA USA; 2 Department of Psychiatry, Boston University Chobanian & Avedisian School of Medicine, Boston, MA USA; 3 Present Address: Department of Psychological and Brain Sciences, Indiana University, Bloomington, IN USA

**Keywords:** Obsessive beliefs, Posttraumatic cognitions, Posttraumatic stress disorder, Obsessive-compulsive disorder

## Abstract

Maladaptive beliefs have been a focus of research in both obsessive-compulsive disorder (OCD) and posttraumatic stress disorder (PTSD). In the OCD literature, beliefs that have typically been studied include inflated responsibility, overestimation of threat, perfectionism, intolerance of uncertainty, and importance and control of thoughts. Meanwhile, in the PTSD literature, negative beliefs about the self, the world, and others have been the focus. We propose that many beliefs commonly studied in the context of OCD research are also relevant to PTSD. Specifically, we propose that trauma may make individuals vulnerable to particular categories of beliefs, whereas other categories may represent pre-existing risk factors. Our theoretical paper highlights how these beliefs have been measured in prior OCD and PTSD research and identifies belief categories that may be clinically relevant but historically under-assessed among individuals with PTSD. Finally, we discuss potential clinical interventions for addressing obsessive beliefs in the context of PTSD treatment.

## Introduction

Theoretical models of obsessive-compulsive disorder (OCD; Rachman, 1997) and posttraumatic stress disorder (PTSD; Foa & Kozak, 1986; A. Wells & Sembi, 2004) both specify maladaptive cognitions as a potential etiological and maintenance factor for the conditions. According to cognitive models of OCD, it is the interpretation of intrusive thoughts as meaningful or dangerous which drives anxiety and leads to compulsive behavior to reduce the anxiety (OCCWG, 1997; Rachman, 1997). Intrusive thoughts are commonly experienced by individuals without OCD, and the content of these thoughts is similar to the content of intrusive thoughts among individuals with OCD (Rachman & de Silva, 1978; Salkovskis & Harrison, 1984), hence the focus on interpretation rather than content. Certain maladaptive beliefs (i.e., obsessive beliefs) are theorized to affect the interpretation of the intrusive thoughts in OCD. For instance, an individual with high intolerance of uncertainty (a category of obsessive beliefs) is less likely to disregard an intrusive thought about whether they turned off their stove, and thus would be more likely to check the stove (i.e., engage in compulsive behavior) in order to reduce their anxiety. Treatment for OCD involves deliberately tolerating situations that evoke intrusive thoughts (i.e., exposures) while abstaining from rituals and/or safety behaviors, as well as cognitively challenging obsessive beliefs that are held (Hellberg et al., 2022).

Maladaptive beliefs are also implicated in theoretical accounts of PTSD. Theories have proposed that maladaptive cognitions about one’s self, others, or the world can contribute to the development and maintenance of PTSD symptoms (Ehlers & Clark, 2000; Foa & Kozak, 1986), and multiple prominent therapeutic interventions involve strategies that are intended to target these cognitive mechanisms. For instance, the rationale behind cognitive processing therapy (CPT) is partially based on the assumptive worlds theory, which posits that individuals hold schemas about the world (e.g., “the world is a safe and just place”) that are not easily shifted. The theory posits that when a traumatic event occurs, individuals must work to create congruency between the new information and their schema which can result in maladaptive beliefs being formed (e.g., self-blame; Janoff-Bulman, 1989; Resick et al., 2017). As such, a key component of CPT is working to shift these beliefs. According to Emotional Processing Theory (EPT), the theory underlying prolonged exposure treatment (PE), fear structures are activated by stimuli associated with the trauma (Foa & Kozak, 1986). EPT posits that PTSD is maintained, at least in part, through maladaptive cognitions (e.g., elevated threat estimation). Adaptive responding involves engaging with trauma related stimuli so that the fear structure is activated and new, competing information can be incorporated (e.g., “*I approached this trauma reminder and nothing bad happened*”).

Within the OCD literature, several different categories of obsessive beliefs have been proposed and a multitude of corresponding measures were developed. In an attempt to reduce the number of measures, harness focus on the most relevant beliefs to OCD, and create more standardization, the Obsessive Compulsive Cognitions Working Group (OCCWG, 1997) identified six different beliefs thought to be most relevant (i.e., overestimation of threat, inflated responsibility, perfectionism, intolerance of uncertainty, importance of thoughts, and the importance of controlling thoughts). They subsequently developed an 87-item measure that assessed these six beliefs called the Obsessional Beliefs Questionnaire (OBQ; OCCWG, 2001). An exploratory factor analysis resulted in a three-factor solution consisting of inflated responsibility/overestimation of threat, perfectionism/intolerance of uncertainty, and importance/control of thoughts; a total of 44 items were retained in the revised scale (the OBQ-44; OCCWG, 2005). Subsequent research has yielded differing factor solutions for the OBQ-44 ranging from a unidimensional structure (Faull et al., 2004) to a four-factor solution (Myers et al., 2008). In their multimethod analysis of the OBQ-44, Moulding et al. (2011) found a four-factor solution (inflated responsibility, overestimation of threat, perfectionism/intolerance of uncertainty, and importance/control of thoughts) and retained 38 items, labeling their measure the OBQ-TRIP. They also introduced a briefer version (the OBQ-20) based on the four-factor solution they had found in order to make the measure more usable by researchers. The authors sought to achieve the highest possible correlations between subscales (which aligned with the four factors identified previously, and had five items apiece) and also the highest possible item-total score correlations, while also having acceptable levels of internal constancy. These categories of beliefs, as measured by various iterations of the OBQ, are elevated in individuals with OCD (Doron et al., 2016; OCCWG, 2001, 2005), but also appear to be heightened in other psychological disorders, alluding to them being transdiagnostic risk factors rather than being specific to OCD (Tolin et al., 2006).

Commonly used measures of maladaptive beliefs in PTSD include the Posttraumatic Cognitions Inventory (PTCI; Foa et al., 1999) and the Posttraumatic Maladaptive Beliefs Scale (PMBS; Vogt et al., 2012). The PTCI includes 33 items assessing three different beliefs, including negative cognitions about the self, negative cognitions about the world, and self-blame, which were determined by an exploratory factor analysis using items generated based on theory and interviews (Foa et al., 1999). However, due to difficulty confirming this factor structure (Whiteman et al., 2022), a 9-item version of the PTCI (with the same subscales) has been developed (Wells et al., 2019) and shown more promising model fit and psychometric properties (Byllesby et al., 2022; Serier et al., 2023). Meanwhile, the PMBS assesses threat of harm, self-worth and judgment, and reliability and trustworthiness of others. The rationale behind measuring these constructs was based upon theory which suggests that trauma may shift these beliefs (McCann et al., 1988). These beliefs, as measured by the PTCI and PMBS, are relevant to PTSD, as indicated by their association with PTSD symptom severity (Held et al., 2017; Vogt et al., 2012). However, obsessive beliefs, as measured by various scales, have also been shown to be associated with posttraumatic stress/PTSD symptoms and elevated in individuals with a diagnosis of PTSD (Banducci et al., 2016; Bardeen et al., 2013; Boelen et al., 2016; Hollingsworth et al., 2018; Nortje et al., 2004; Raines et al., 2019; Tyler et al., 2021). Furthermore, exposure to trauma may make one vulnerable to these beliefs, as the amount of potentially traumatic events that one has experienced is positively associated with the three subscales of the OBQ-44 (Fenlon, 2022), and childhood abuse is positively associated with obsessive beliefs (Briggs & Price, 2009). Thus, it appears that the existing PTSD-specific measures of maladaptive beliefs may not fully capture the range of beliefs that are associated with PTSD symptoms or may emerge after experiencing trauma.

Emerging research has found that trauma exposure can make individuals vulnerable to developing OCD (Pinciotti & Fisher, 2022; Reifels et al., 2019), and has also found that trauma and PTSD is linked to more severe OCD (Ojserkis et al., 2017; Pinciotti et al., 2022). Given this research, it is interesting to consider the overlap between these disorders as it pertains to cognitive mechanisms (Despotes et al., 2021), including commonalities and differences. In this paper we seek to examine theoretical links between obsessive belief categories and PTSD and suggest areas for future inquiry. Given the paucity of research on obsessive beliefs and PTSD, a systematic review would not be possible at this stage. However, we review a wide range of theoretical and empirical literature across multiple disciplines to examine how some of these obsessive beliefs may, in part, develop from traumatic and/or stressful events and how these beliefs may serve to sustain PTSD symptoms. Additionally, we examine the overlap between measures commonly used in OCD research (e.g., the OBQ-20) and those used in PTSD research (e.g., the PTCI), and explore differences between the measures which may indicate a need for future research and measurement development focused on obsessive beliefs in PTSD. It is possible that current conceptualizations and measurement of posttraumatic cognitions are too narrow and miss additional cognitive vulnerabilities, which in turn could have important clinical and research implications.

## Overestimation of Threat

Overestimation of threat can be defined as an individual’s tendency to assign unrealistically heightened probabilities of something harmful or disastrous happening. This general schema impacts how contexts are appraised in terms of their safety. Although ample research has found a relationship between overestimation of threat and OCD symptoms specifically (OCCWG, 2003), experimental and cross-sectional work has alluded to its relevancy to a wide range of anxiety-based disorders. For example, one study found that individuals with panic disorder were more likely than healthy control subjects to estimate a higher probability of a negative outcome (i.e., a shock) following presentation of a threatening stimuli (Wiedemann et al., 2001). Another study found a positive association between overestimation of threat and health anxiety symptoms, and found that overestimation of threat mediated the relationship between illness-related intrusive thoughts and health anxiety symptoms (Arnáez et al., 2021). Taken together, overestimation of threat appears to be relevant to a range of anxiety-based disorders. Unsurprisingly, overestimation of threat has been implicitly implicated in a number of theoretical accounts of PTSD (Chemtob et al., 1988), however, the terminology used varies with “threat expectancy” (Chemtob et al., 1988), “the world is entirely dangerous” (Foa & Rothbaum, 1998), and “safety beliefs” (Resick et al., 2017) being used to describe a similar concept. In PTSD, this construct may be most closely tied to the *negative cognitions about the world* subscale of the PTCI and the *threat of harm* subscale of the PMBS (Foa et al., 1999; Vogt et al., 2012).

A comparison of the items assessing these similar constructs suggests some overlap in that across scales it is implied that negative or catastrophic events are high-probability incidents (see Table 1). The items for the subscales of the PTCI and PMBS more explicitly assess the loss or lack of trust in others (Foa et al., 1999; Vogt et al., 2012), whereas the subscale for the OBQ-20 appears to target loss or lack of trust in one’s self. This distinction is important because both loss/lack of trust in others and the self are theorized as potential mechanisms linking traumatic exposure to the development of PTSD (Foa et al., 1999; McCann et al., 1988). It is worth noting the PMBS (e.g., *self-worth and judgment*: “I don’t feel confident that I can make good decisions for myself”) and PTCI (e.g., *negative cognitions about self*: “I can’t trust that I will do the right thing”) have items that include content related to trust (or the lack there of) in one’s self, but these are general items rather than being specifically linked to traumatic/catastrophic events. At the subscale level, *threat of harm* (a subscale of the PMBS) has a moderately positive association with PTSD symptoms (Vogt et al., 2012), and *negative cognitions about the world* (a subscale of the PTCI) has a mostly moderate to strong association with PTSD symptoms (Blain et al., 2013; Foa & Rauch, 2004; Held et al., 2017; Horwitz et al., 2018; Renaud, 2008). Some research has found that *threat of harm* and *negative cognitions about the world* mediate symptom change (Kumpula et al., 2017; Scher et al., 2017), although other research has not found that cognitive belief change temporally precedes symptom change (Held et al., 2022; Lee et al., 2021). While these conflicting results deserve further inquiry, the scales which appear to be most closely aligned with overestimation of threat are positively related to higher PTSD symptoms (Held et al., 2017; Vogt et al., 2012) and there is some evidence that changes in these beliefs predicts future PTSD symptoms (Kumpula et al., 2017; Scher et al., 2017).

**Table 1:** Example items assessing obsessive belief domains in the OBQ-20, PTCI, and PMBS

Belief domain	Measure		
	OBQ-20	PTCI	PMBS
Overestimation of Threat	“If I do not take extra precautions, I am more likely than others to have or cause a serious disaster.” “I am more likely than other people to accidentally cause harm to myself or to others.”	“The world is a dangerous place.” “You can never know who will harm you.”	“I avoid other people because they might hurt me” “I don’t trust anyone anymore”
Inflated Responsibility	“If I don’t act when I foresee danger, then I am to blame for consequences.” “For me, not preventing harm is as bad as causing harm.”	“The event happened because of the way I acted.” “Somebody else would have stopped the event from happening.”	-
Perfectionism	“I should be upset if I make a mistake.” “I must keep working until it’s done exactly right.”	-	-
Intolerance of Uncertainty	-	-	-
Importance of Thoughts	“For me, having bad urges is as bad as actually carrying them out.” “Having bad thoughts means I am weird or abnormal.”	-	-
Importance of Controlling Thoughts	“Having intrusive thoughts means I’m out of control.”	“If I think about the event, I will not be able to handle it.”	-

Note. OBQ-20 = Obsessive Beliefs Questions – 20 item version; PTCI = Posttraumatic Cognitions Inventory; PMBS = Posttraumatic Maladaptive Beliefs Scale

**Fig. 1:**
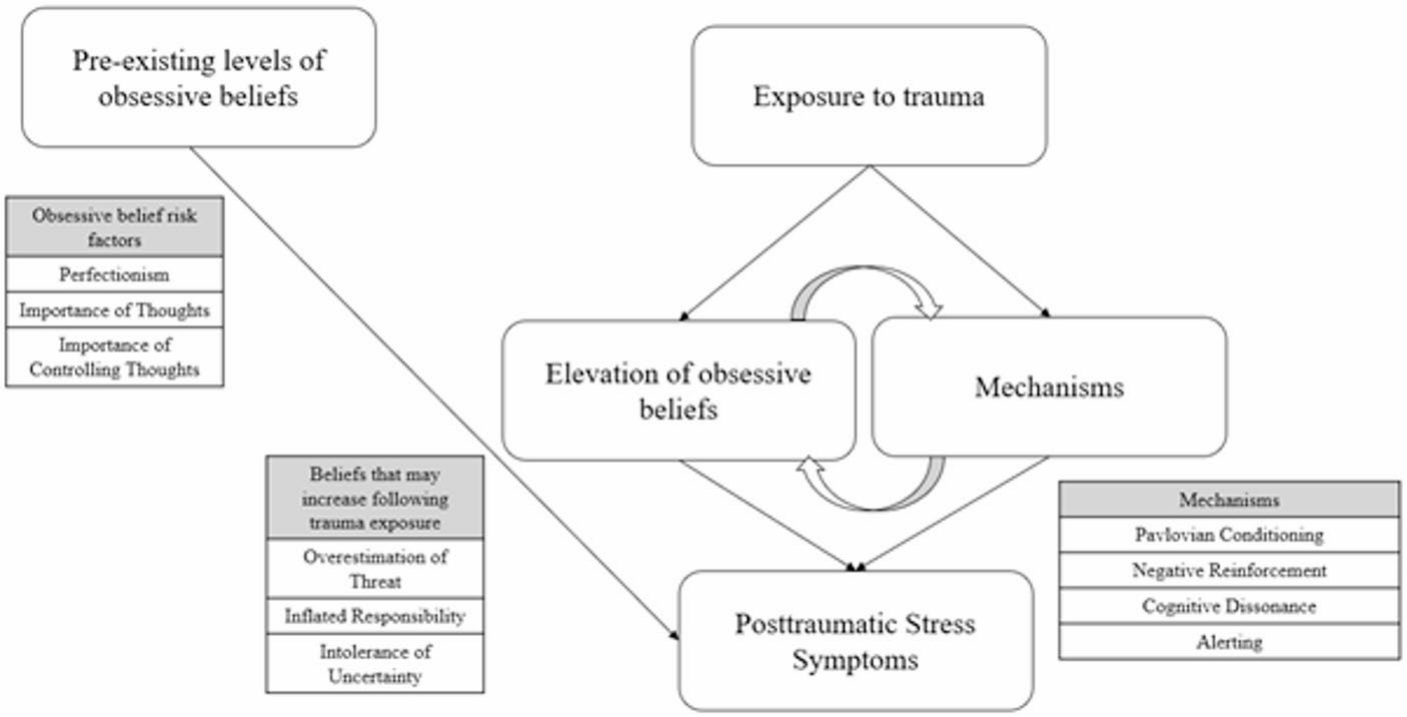
Schematic showing how both pre-existing levels of obsessive beliefs and beliefs elevated by trauma (through various mechanisms) contribute to PTSD symptoms

Synthesis of the literature suggests a pathway by which traumatic and/or stressful events may lead to an increase in overestimation of threat. The first component of this pathway is based on the fear conditioning literature. According to Pavlovian theory, a conditioned stimulus (CS; i.e., an innocuous/neutral stimuli) is paired with an unconditioned stimulus (UCS; i.e., a stimulus which elicits a physiological response on its own). When the CS-UCS pairing is sufficiently strong, the CS begins to elicit the response even without the presentation of the UCS. For example, a soldier deployed to a combat zone may begin to pair things such as trash and crowds of people (i.e., CS) with outcomes such as an IED explosion or a terrorist attack (i.e., UCS). Importantly, conditioning to aversive stimuli remains relatively intact despite changes in context (Bouton & King, 1983; Hall & Honey, 1989) and the passage of time (Gleitman & Holmes, 1967). Thus, when the soldier returns home, the presence of trash on the side of the road or crowds of people may still produce fear. The second component of this pathway is based on evolutionary theory. That is, it is believed that in the Pleistocene, humans developed a fear structure which allowed them to adaptively respond to threatening situations (e.g., the presence of a predator) in order to increase their chances of survival (Bereczkei, 2000; Öhman & Mineka, 2001). Furthermore, adaptive responding may include responding to cues (e.g., sounds, smells, visual stimuli) that alert the individual to potential threat (Öhman & Mineka, 2001). Interestingly, individuals may tend to project harmful, rather than positive or neutral, characteristics when information about an entity is unknown (Liang et al., 2022). In an experiment by Liang et al. (2022), participants were presented with a vignette about a biologist who discovered novel species and were given information and reported the likelihood that harmful, positive, or neutral properties were held by that species and other novel species discovered by the biologist. Results revealed that participants were more likely to project harmful qualities to a different species. In our example of the soldier who is deployed, it is easy to see how it was adaptive to overgeneralize cues to an aversive event, because failing to identify an IED or incoming attack could mean the loss of life (see Chemtob et al. (1988) for a discussion on the initial adaptive nature of certain PTSD symptoms in combat zones). The behaviors that were once adaptive become negatively reinforced as avoiding or taking precautions would ostensibly result in the absence of a negative outcome. Put differently, overestimation of threat may be conditioned due its earlier adaptive properties (see Fig. 1 for a depiction).

Overestimation of threat is also likely to play a role in the maintenance of PTSD symptoms. Overestimation of threat has shown a positive association with intrusion based symptoms (Nortje et al., 2004), which is not surprising given that overestimation of threat is likely to lead to an increased number of trauma cues. Increased physiological reaction to trauma cues would also likely occur due to overestimation of threat as experimental studies have found that one’s expectancy for adverse outcomes, when presented with fear-relevant stimuli, is predictive of autonomic responding even with specific instruction that the aversive event will not happen (Davey, 1992). Since the general schema of overestimating threat will apply to a wide range of ambiguous situations, individuals with heightened overestimation of threat may be more likely to avoid these situations, which as discussed above may have been adaptive in certain contexts but will ultimately lead to less inhibitory learning (i.e., CS-no UCS). Similarly, hypervigilance may be increased due to the heightened probability assigned to a range of contexts. That is, hypervigilance may be used as a coping mechanism to counteract any potential threats.

## Inflated Responsibility

Inflated responsibility is a belief where individuals hold an excessive sense of responsibility for outcomes, meaning that they inappropriately assume all or inordinate amounts of responsibility for either (preventing) negative outcomes or (producing) positive outcomes. This belief has shown a consistent association with OCD symptoms (OCCWG, 2003), but also appears to be relevant to a wide range of mental health conditions (Tolin et al., 2006), including social anxiety disorder (Jones & Rakovshik, 2019), eating disorders (Kerr et al., 2016; Lavender et al., 2006), and generalized anxiety disorder (Gústavsson et al., 2021). Conceptually, inflated responsibility is most closely aligned with the *self-blame* subfactor of the PTCI. The similarity between *self-blame* as measured by the PTCI and inflated responsibility as measured by the OBQ-20 is that both implicate the self as responsible for traumatic or negative events. The difference between the two may be that the *self-blame* subscale appears to be assessing post-hoc reasoning for why a traumatic event occurred (e.g., “The event happened because of the way I acted”), whereas the *inflated responsibility* subscale appears to be measuring beliefs about responsibility for events that may happen in the future (e.g., “If I don’t act when I foresee danger, then I am to blame for consequences”). Assessing for beliefs regarding future events may be important since future episodic thinking relies on similar neuroanatomical regions and cognitive processes as autobiographical memories (Schacter et al., 2008), and thus may have a tendency to restrict thought content generated for future events (Brown et al., 2016; Schacter & Addis, 2007). For instance, in a study of combat veterans with and without PTSD, veterans with PTSD tended to recall more combat-related content when imagining future events than veterans without PTSD (Brown et al., 2013). It is also important to note that individuals may have a tendency to project worse posttraumatic stress symptoms for imagined future events in contrast to past events that happened (Rubin, 2014). Taken together, assessing current/future, in addition to past, beliefs regarding responsibility may be especially important for those who have experienced trauma or posttraumatic stress symptoms. It seems plausible that those who have heightened past as well as current/future beliefs about responsibility may be particularly vulnerable.

Extant theory on inflated responsibility postulates that a catastrophic or negative event where one’s actions or inactions are deemed to have contributed to the outcome can lead to subsequent increases in inflated responsibility (Salkovskis et al., 1999). This process seems to closely parallel the development of moral injury by which emotional distress can occur due to causing, not preventing, or witnessing an event that is counter to one’s moral values or expectations (Litz et al., 2009). Certain professions (e.g., healthcare workers, service members) may be especially vulnerable to developing inflated responsibility since not only is high exposure to trauma and stress common but critical outcomes may be dependent upon the actions that the individual takes. Preconceived ideals of their role developed through media representations, employer communication, and explicit statements (e.g., the Hippocratic Oath) may serve to heighten the individual’s perceived responsibility. When an action or inaction leads to a consequential negative outcome (e.g., death of a patient) then responsibility may be particularly inflated, perhaps through cognitive dissonance which is also a proposed mechanism of moral injury (Litz et al., 2009). Furthermore, individuals in professions such as healthcare or the armed forces may take on the role of protector (Bacon, 2017), which may be difficult to turn off in other contexts (e.g., with family). For instance, some combat veterans may develop a survivalist identity in which they take pride in their ability to manage life-threatening events (Glover, 1988).

As it relates to symptom maintenance in PTSD, inflated responsibility may lead to hypervigilance and avoidance being used to manage difficult emotions. In individuals with OCD, inflated responsibility is linked to checking symptoms (a behavioral strategy to ensure that one is not responsible for a catastrophic event; Arntz et al., 2007; OCCWG, 2001). Similarly, individuals with PTSD may use hypervigilance and avoidance to counter the inflated sense of responsibility (and corresponding distress) they feel or, in the case of avoidance, to ensure they are not responsible for any traumatic event.

## Perfectionism and Intolerance of Uncertainty

Perfectionism is a multi-faceted construct with varying definitions (Frost et al., 1990; Hewitt & Flett, 1991), however, as it relates to its conceptualization within OCD, perfectionistic beliefs regarding heightened concern over mistakes and lower perceived ability to complete tasks appear to be most relevant (Frost & Steketee, 1997). There is some evidence of a relationship between perfectionism and posttraumatic stress symptoms in the existing literature. For instance, in a community sample of individuals who had experienced a traumatic event, socially-prescribed perfectionism (i.e., perfectionistic standards that are held due to the belief that important people in their life expect them to be perfect; Hewitt & Flett, 1991) was found to be positively linked to posttraumatic stress symptoms but only for those who also held low perceived sense of control (Molnar et al., 2021). In another study, perfectionistic cognitions were related to higher posttraumatic stress symptoms even after controlling for potential covariates (e.g., anxiety sensitivity) in a treatment seeking sample (Tyler et al., 2021). In a latent profile analysis using an undergraduate sample, Christian et al. (2021) identified groups that were high in perfectionism, high in impulsivity, high in both, and low in both. They found that groups high in perfectionism and high in both perfectionism and impulsivity had the highest associations with posttraumatic stress symptoms. Finally, in a clinical sample of individuals who had experienced a sexual trauma, perfectionism had a strong positive association with PTSD symptoms (Egan et al., 2014). These studies employed various measures of perfectionism, including the Hewitt and Flett Multidimensional Perfectionism Scale (Hewitt & Flett, 1991), the Perfectionism Cognitions Inventory (Flett et al., 1998), the Frost Multidimensional Perfectionism Scale (Frost et al., 1990), and the Clinical Perfectionism Questionnaire (Fairburn et al., 2003). However, the associations found were generally small to moderate in size, with the exception of Egan et al. (2014) who found strong associations between the *concerns over mistakes* subscale of the Frost Multidimensional Perfectionism Scale and the Clinical Perfectionism Questionnaire, and posttraumatic stress symptoms. Thus, there appears to be some overlap in that *concerns over mistakes* may be relevant to both OCD and PTSD. As it relates to specific PTSD symptoms, perfectionism may be especially relevant to intrusion symptoms given that perfectionism has an empirically established link with rumination (Abdollahi, 2019; O’Connor et al., 2007), and intrusive and ruminative thoughts have many similarities. Unfortunately, the studies described here did not examine associations between perfectionism and specific symptom clusters of PTSD, such as intrusions, so further research will be needed to determine if particular clusters show greater or lesser associations with perfectionism.

It is plausible that traumatic and/or stressful events could lead to heightened levels of perfectionism. According to the assumptive worlds theory (Janoff-Bulman, 1989), individuals make assimilated attributions to fit pre-existing beliefs about the world. For example, an individual who experiences a sexual trauma may look for internal attributions for why the event happened so that they can maintain their pre-existing beliefs (e.g., “my partner loves me, they wouldn’t rape me”). If an individual makes assimilation attributions regarding self-blame for traumatic events, then that individual may begin to scrutinize their actions and develop a heightened sense of perfectionism with a particular focus on avoiding any potential “mistake.”

Of the obsessive beliefs reviewed here, intolerance of uncertainty has received the most attention in the PTSD literature. Intolerance of uncertainty has been shown to have a consistent link with posttraumatic stress symptoms across a range of samples (Arbona et al., 2022; Raines et al., 2019; Shapiro et al., 2022). The short-form version of the Intolerance of Uncertainty Scale (IUS; Carleton et al., 2007) is often used to measure the construct. The IUS contains two subscales: *prospective anxiety* (i.e., distress regarding uncertain future events) and *inhibitory anxiety* (i.e., uncertainty leading to behavioral inhibition). Both prospective and inhibitory anxiety have demonstrated mechanistic links which may sustain PTSD symptoms. Prospective anxiety was found to be related to alerting (i.e., a part of the attentional system which functions to sustain vigilance toward stimuli; Posner & Rothbart, 2007) in a sample of undergraduates after controlling for state anxiety (Fergus & Carleton, 2016). Meanwhile, higher levels of inhibitory anxiety predict future posttraumatic stress symptoms (Boelen, 2019; Boelen et al., 2016). Hollingsworth et al. (2018) reasoned that heightened feelings of uncertainty may lead to avoidance of activities. It is also possible that some of the mechanisms described in the section on overestimation of threat are relevant. For example, intolerance of uncertainty may be adaptive in certain contexts and may be behaviorally reinforced. As it relates to specific symptom clusters, higher levels of negative alterations in cognition and mood along with alterations in arousal and reactivity were found to be linked to higher intolerance of uncertainty after controlling for anxiety sensitivity and college stress in a sample of Latina college women who had experienced a traumatic event (Arbona et al., 2022). Other work suggests that the relationship between intolerance of uncertainty and symptom clusters may partially depend on the type of trauma experienced, as Shapiro et al. (2022) found that individuals who had experienced a sexual trauma, intolerance of uncertainty was linked to every symptom cluster with the exception of intrusion symptoms, whereas for individuals who had experienced a non-sexual trauma, intolerance of uncertainty was only linked to alteration in arousal and reactivity. As these results collectively suggest, intolerance of uncertainty appears to be a robust predictor of posttraumatic stress symptoms, and important progress has been made in identifying pathways by which these beliefs may sustain PTSD symptoms. Measurement of the construct remains somewhat challenging, however. As noted above, much of the prior work has relied upon the IUS as the measure of intolerance of uncertainty. Although one of the identified factors of the OBQ-20 is labeled *perfectionism/intolerance of uncertainty*, the subscale has low face validity as all of the associated items appear to be measuring perfectionism alone (e.g., “To be a worthwhile person, I must be perfect at everything I do”, “For me, things are not right if they are not perfect”). As for the existing PTSD-specific cognitive scales, neither the PTCI nor the PMBS include items relevant to this category of belief.

## Importance of Thoughts and Controlling Thoughts

The importance of thoughts and the need to control them are two additional obsessive beliefs that have been shown to be related to OCD (OCCWG, 2003). Past research has found that individuals with OCD have higher levels of these beliefs than an anxiety control group, and in turn the anxiety control group had higher levels of these beliefs than healthy controls (Tolin et al., 2006). A subcomponent of importance and control of thoughts called thought-action fusion is not specific to OCD (Berle & Starcevic, 2005) and has been linked to a range of psychological disorders, including eating disorders (Coelho et al., 2012), depression (Gjelsvik et al., 2018), generalized anxiety disorder (Thompson-Hollands et al., 2013), and social anxiety disorder (Hezel et al., 2019). Thought-action fusion occurs when an individual believes that having a thought (e.g., “I am going to die in a car accident”) increases the chances of those thoughts actually happening in real life (i.e., thought-action fusion-likelihood), or is morally the same (e.g., thinking something unkind about someone is the same as being unkind to them) as actually doing a particular action (i.e., moral thought-action fusion) (Shafran et al., 1996).

Importance and control of thoughts may be relevant to the sustainment of intrusion symptoms in PTSD. Empirically, importance and control of thoughts has been shown to be predictive of intrusive thinking (Wahl et al., 2020). If individuals who have experienced a traumatic event or have posttraumatic stress symptoms/PTSD believe that thoughts about the event must be controlled then they may have a tendency to engage in maladaptive thought control strategies (e.g., thought suppression). These maladaptive control strategies have the unintended consequence of actually increasing the frequency of intrusive thoughts (Shipherd & Beck, 1999, 2005). Further research has shown that the attributions that one makes about the recurrence of intrusive thoughts following suppression attempts is predictive of distress and frequency of intrusive thoughts (Magee & Teachman, 2007), which could be especially relevant to PTSD in that individuals with this disorder sometimes report feeling that their intrusions are signs that they are going “crazy” (Ehlers & Steil, 1995; Steil & Ehlers, 2000). These fears may provoke further efforts to engage in (ineffective) mental avoidance strategies, thus perpetuating the intrusions. It may be the case that beliefs about importance and control of thoughts are not necessarily generated by experiencing a traumatic event, but are rather pre-existing response styles that confer risk for developing and maintaining PTSD symptoms (Ehlers & Clark, 2000; Ehlers & Steil, 1995).

The OBQ-20 has items assessing moral thought-action fusion (e.g., “Having a bad thought is morally no different than doing a bad deed”) but not thought-action fusion-likelihood. Thus, the ability to fully assess the construct of thought-action fusion will be limited if the OBQ-20 is used. Researchers interested in studying all components of thought-action fusion may have to consider alternative measures. The OBQ-44 and OBQ-Trip each have just one item assessing thought-action fusion-likelihood, which may be insufficient to measure this more narrow form of belief. A separate, thought-action fusion specific measure (the Thought-Action Fusion Scale; Shafran et al., 1996) has been developed, however most of the content is geared toward obsessional concerns (e.g. “When I think about making an obscene remark or gesture in church, it is almost as sinful as actually doing it”). The *Internal State and Thought-Action Fusion* subscale of the Illusory Beliefs Inventory (Kingdon et al., 2012), has content non-specific items assessing thought-action fusion-likelihood but not moral thought-action fusion. Thus, researchers will have to carefully consider whether they are interested in importance and control of thoughts more generally or specific facets and choose the best measure(s) accordingly.

## Discussion

In this paper we have reviewed the relevance of commonly studied obsessive beliefs to PTSD and presented a summary of existing empirical findings for each. We sought to examine which beliefs may increase as a result of exposure to trauma or stressful events. Overestimation of threat and inflated responsibility appear to have the strongest theoretical rationale for being vulnerable to increasing following a traumatic or stressful event, with some potential evidence for intolerance of uncertainty increasing as well. The theoretical rationale for trauma leading to increases in perfectionism appears somewhat less convincing, which may not be surprising given that perfectionism is largely thought to develop as a result of parenting styles and criticism (Frost et al., 1991). Meanwhile, beliefs surrounding the importance and control of thoughts appear more likely to be pre-existing risk factors which can contribute to the maintenance of PTSD symptoms through their tendency to elicit certain behavioral responses (e.g., thought suppression).

We have further considered the measurement of these obsessive beliefs as it relates to PTSD. The *overestimation of threat* subscale for both the OBQ-TRIP and OBQ-20, *threat of harm* subscale of the PMBS, the *negative cognitions about the world* of the PTCI, all appear to capture a similar construct, with the *overestimation of threat* subscale being less focused on loss of trust in others as it relates to threat. When assessing responsibility beliefs, it may be advantageous to use both the *inflated responsibility* subscale as measured by the OBQ-Trip or OBQ-20 as well as the *self-blame* subscale of the PTCI, as this combination would provide measures of responsibility related to past (*self-blame*) and current/future (*inflated responsibility*) events. There is a plethora of perfectionism scales that are comprised of varying subscales (see Lo et al., 2020 for a review of multidimensional perfectionism measures). Although the appropriate measure will largely depend on the research question, it would appear as though heightened concerns about mistakes may be more relevant to PTSD than some of the other dimensions of perfectionism such as *self-* or *other-oriented perfectionism*. The short-form IUS has been used often as a measure of intolerance of uncertainty and both its subscales (*prospective anxiety* and *inhibitory anxiety*) appear to be relevant to PTSD. As previously stated, the *perfectionism/intolerance of uncertainty* subscale of the OBQ-TRIP and OBQ-20 would not be an appropriate measure of intolerance of uncertainty given the low face validity. Finally, the *importance/control of thoughts* subscales of the OBQ-TRIP, OBQ-44, or OBQ-20 can be used by researchers who are interested in the construct at a general level. Those interested in more specific facets of importance and control of thoughts, such as thought-action fusion-likelihood, may consider alternative measures.

## Implications

It is possible that existing treatments such as CPT or PE may reduce the obsessive beliefs reviewed here. For instance, CPT explicitly focuses on challenging inappropriate beliefs about responsibility/blame for a traumatic event, among other trauma-related cognitions. More broadly, the cognitive restructuring skills gained through CPT may translate to the skills naturally being adopted in situations where beliefs such as intolerance of uncertainty or thought-action fusion are activated, although this possibility requires empirical investigation. PE, or other exposure-based interventions, may also similarly lead to reductions in obsessive beliefs. In particular, overestimation of threat may be reduced in that the feared stimuli (i.e., the CS) will ostensibly not produce the UCS, thus leading to an expectancy violation which may reduce threat perceptions. It is important to note that according to inhibitory learning theory, the CS-UCS link remains intact despite disconfirming information (Bouton, 1993) and thus supplemental intervening (see Craske et al., 2008, 2014 for recommendations on enhancing inhibitory learning) to reinforce the CS-no UCS link will likely enhance reductions in beliefs such as overestimation of threat. Both CPT and PE have been shown to reduce posttraumatic cognitions (Held et al., 2022; Zalta et al., 2014); although the particular categories of obsessive beliefs reviewed above have not been specifically tested in the context of these treatments. As we have reviewed, there is overlap between maladaptive posttraumatic cognitions and obsessive beliefs. Obsessive beliefs have also been found to decrease as a result of cognitive and behavioral interventions (Adams et al., 2012; Wilhelm et al., 2005) that share putative mechanisms of action with PTSD interventions (e.g., completing exposures).

The malleability of these obsessive beliefs may depend upon how long they have been held and the degree to which these beliefs are thought of as irrational by the patient. Past research has found that more time since trauma exposure is associated with less improvement in posttraumatic cognitions, with the effects being more pronounced for individuals completing CPT (which focuses on directly challenging trauma-related thoughts) as opposed to written exposure therapy (which does not include any instruction to challenge trauma-related beliefs) (Cole et al., 2024). One possibility suggested by the authors is that these beliefs may become more ingrained through behavioral reinforcement over time. Another determinant of the malleability of these beliefs may be the degree to which they are considered irrational by the individual. Overvalued ideation occurs when symptoms or beliefs are thought of as being rational (Foa, 1979; Neziroglu & Mancusi, 2014). For example, in the context of overestimation of threat, a patient may state that they know their beliefs about threat are unrealistic and that there is not in reality such a likely probability of fears coming to fruition (absence of overvalued ideation) or may hold with conviction that their fears about threat are realistic and relay evidence supporting their beliefs (overvalued ideation). Certain patients (e.g., those with a military background) may feel that they were trained to think in a particular way (e.g., overestimating threat, not tolerating uncertainty) and may view these styles of thinking as adaptive and thus be more likely to have overvalued ideation. Obsessive beliefs in the absence of overvalued ideation may be more amenable to common interventions such as cognitive restructuring. Importantly, individuals with OCD tend to respond more poorly to treatment when they have higher levels of overvalued ideation (Veale, 2007).

It is worth noting that there is evidence that PTSD symptoms can be reduced even when there is a smaller reduction in posttraumatic cognitions (Cole et al., 2024; Sloan et al., 2018), but it raises concerns as to whether an individual can be considered “healthy” or a treatment success while continuing to express high levels of these cognitions or beliefs. If these beliefs are behaviorally ingrained, are thought of as perfectly rational, or do not dissipate following commonly used PTSD treatments, what approaches might be effective to reduce their influence? One possibility is to explicitly highlight these beliefs during exposure therapy. For instance, overestimation of threat and intolerance of uncertainty may be optimal targets of expectancy violation during exposure. By assessing pre-, during, and post-exposure ratings of threat or perceived ability to tolerate uncertainty, a therapist can highlight discrepancies (e.g., “*you thought there was an 80% chance that you or someone else would get seriously injured*,* but it looks like no one got hurt at all*”). Inflated responsibility may be responsive to treatments such as Adaptive Disclosure, which involves engaging in an imaginal conversation about a traumatic (i.e. morally injurious) event with a moral authority who is understanding and forgiving (Gray et al., 2021; Litz et al., 2016). If the individual’s sense of inflated responsibility is believed to stem from a morally injurious event, then the Adaptive Disclosure intervention may not need to be adjusted. In other cases, the intervention could be altered to focus on the patient’s perceived responsibility with a figure who is caring, accepting, and offers alternative perspectives. These suggestions come with the caveat that they have not been empirically tested, and thus evaluations of if, and for whom, they are effective will be needed.

## Future Directions

In the present overview, we have sought to explore the potential relevance of obsessive beliefs to PTSD. We suspect that, based on both theoretical models of PTSD and the preliminary empirical evidence presented here, these beliefs play a role in the development and/or maintenance of PTSD symptoms. However, further investigation is needed to determine the nature of such a relationship, and whether the belief domains described here demonstrate distinct patterns with regard to symptom development or severity. It will also be important to examine the relationship between obsessive beliefs and PTSD symptoms across a range of samples (e.g., among individuals of different genders/racial categories, or with different types of pre-trauma life experiences). It appears that the relationship between specific beliefs and symptoms is dependent upon the type of trauma experienced (e.g., Shapiro et al., 2022) and demographic factors (e.g., Kim et al., 2022). If such a relationship is found to exist, then logical next steps include examining whether these obsessive beliefs change as a result of existing treatments for PTSD and whether such change predicts symptom improvement. Longitudinal analyses examining the sequence of change in posttraumatic cognitions/obsessive beliefs and symptoms have both produced conflicting findings, with work finding that cognition change precedes symptom change (Kumpula et al., 2017), concurrent change (Lee et al., 2021), and a reciprocal relationship (Falkenstein et al., 2020; Held et al., 2022). Part of the reason for the discrepant findings may be that in many intervention research studies the frequency of measurement is not sensitive enough to adequately capture these temporal patterns. Another potential problem is that while posttraumatic cognitions and obsessive beliefs are posited as risk factors or mechanisms that can lead to the development of PTSD or OCD, they are also symptoms themselves. Criterion D of the diagnosis of PTSD includes symptoms such as negative beliefs about oneself and inappropriate beliefs about blame for the traumatic event (American Psychiatric Association, 2013). In the case of OCD, it is difficult to disentangle intrusive thinking (i.e., obsessions) from obsessive beliefs. To illustrate this with an example, imagine an individual having recurrent obsessions about harm happening to their loved ones; that person would likely have fairly high levels of overestimation of threat, and the overestimation of threat would be closely linked to their intrusive thoughts. At what point does overestimation of threat become a proxy for the obsession? We would argue that in this case the change in the intrusive thinking, or obsessions, would happen essentially concurrently with changes in the obsessive belief. Thus, we suggest that researchers think carefully about the projected time course between belief change and symptom change. It may be the case that the temporal relationship depends on the specific symptoms being examined, with belief change sometimes preceding symptom change and in other cases changing concurrently.

## Conclusion

In this paper we have reviewed the relevancy of obsessive beliefs to PTSD. Our review of the literature suggests that there is overlap between obsessive beliefs studied primarily in OCD and posttraumatic cognitions. Some beliefs may increase as a result of traumatic experiences while many of these beliefs may sustain symptoms. Careful selection of measures and research design will help elucidate the relationship between obsessive beliefs and PTSD symptoms.

## Data Availability

There is no data associated with this manuscript.
